# Improved phosphoproteomic analysis for phosphosignaling and active-kinome profiling in Matrigel-embedded spheroids and patient-derived organoids

**DOI:** 10.1038/s41598-018-29837-1

**Published:** 2018-07-30

**Authors:** Yuichi Abe, Asa Tada, Junko Isoyama, Satoshi Nagayama, Ryoji Yao, Jun Adachi, Takeshi Tomonaga

**Affiliations:** 1 Laboratory of Proteome Research, National Institute of Biomedical Innovation, Health and Nutrition, Ibaraki, Osaka 567-0085 Japan; 2 Laboratory of Proteomics for Drug Discovery, Center for Drug Design Research, National Institute of Biomedical Innovation, Health and Nutrition, Ibaraki, Osaka 567-0085 Japan; 3 Department of Gastroenterological Surgery, Cancer Institute Hospital, Japanese Foundation for Cancer Research, 135-8550 Tokyo, Japan; 4 Division of Cell Biology, Cancer Institute, Japanese Foundation for Cancer Research, 135-8550 Tokyo, Japan

## Abstract

Many attempts have been made to reproduce the three-dimensional (3D) cancer behavior. For that purpose, Matrigel, an extracellular matrix from Engelbreth-Holm-Swarm mouse sarcoma cell, is widely used in 3D cancer models such as scaffold-based spheroids and patient-derived organoids. However, severe ion suppression caused by contaminants from Matrigel hampers large-scale phosphoproteomics. In the present study, we successfully performed global phosphoproteomics from Matrigel-embedded spheroids and organoids. Using acetone precipitations of tryptic peptides, we identified more than 20,000 class 1 phosphosites from HCT116 spheroids. Bioinformatic analysis revealed that phosphoproteomic status are significantly affected by the method used for the recovery from the Matrigel, i.e., Dispase or Cell Recovery Solution. Furthermore, we observed the activation of several phosphosignalings only in spheroids and not in adherent cells which are coincident with previous study using 3D culture. Finally, we demonstrated that our protocol enabled us to identify more than 20,000 and nearly 3,000 class 1 phosphosites from 1.4 mg and 150 μg of patient-derived organoid, respectively. Additionally, we were able to quantify phosphosites with high reproducibility (r = 0.93 to 0.95). Our phosphoproteomics protocol is useful for analyzing the phosphosignalings of 3D cancer behavior and would be applied for precision medicine with patient-derived organoids.

## Introduction

Reconstruction of cancer behavior in tissues is important for comprehensive understanding of cancer biology. Thus, many researchers have attempted to construct three-dimensional (3D) cancer models using cancer cell lines and patient tissues, termed spheroids and organoids, respectively^1^. Several methods are known for generating cancer spheroids (with or without scaffold supports) that allow the analysis of cancer in 3D behavior and their cell-matrix interactions^2,3^. An advantage of scaffold-based spheroids is that they enable cancer cells to respond to stimulation from extracellular signaling owing to signal transduction through the interactions of transmembrane proteins with integrin-binding ligands in the scaffolds^2^. Recently, organoid culture systems that partially mimic the 3D architecture of specific organs have been developed. These systems have been rapidly adopted by researchers working on many types of cancers, including stomach, liver, pancreas, colon, and kidney^4,5^. As a result, comprehensive molecular profiling of cancer organoids using genomic, transcriptomic, and proteomic data has contributed to the identification of novel driver genes and molecular-level characterization of these cancers^6–8^.

Proteomic data is important for screening novel drug targets because most therapeutic targets are proteins. Kinases, which are enzymes that mediate growth signaling pathways, have been identified as notably druggable targets^9^. Accordingly, our group has previously identified novel targets for Cetuximab-resistant colorectal cancer by profiling kinome activity using deep phosphoproteomic analyses of cancer cell lines^10^. However, the two-dimensional (2D) cell cultures do not perfectly reflect the actual environments of cancer tissues in several respects, such as the interactions with extracellular matrices that occur in 3D cultures^1^. Therefore, phosphoproteomic analysis of 3D culture systems such as scaffold-based spheroids and organoids is necessary for accurate profiling of active kinome in cancer tissues under 3D conditions.

Matrigel, an extracellular matrix secreted from Engelbreth-Holm-Swarm mouse sarcoma cell, is widely used for preparing scaffold-based spheroid or organoid cultures. However, proteomic analysis of Matrigel-embedded samples presents difficulties in terms of sample preparation because of contaminants in Matrigel that hamper LC-MS/MS identification of peptides through ion suppression effects. Consequently, removal of small compounds and insoluble materials has been conducted with SDS-PAGE or centrifugation of tryptic peptides to facilitate proteomic analysis of Matrigel-embedded samples^11^. However, it remains difficult to perform phosphoproteomic analysis of Matrigel-embedded samples because the contaminants cannot be removed efficiently by conventional methods based on in-solution digestion.

Therefore, in this study we have sought to establish a novel protocol for efficiently removing the contaminants present in Matrigel. Because precipitation of proteins with organic solvents has also been applied to the removal of contaminants such as lipids or DNA^12^, we applied acetone precipitation of digested peptides to phosphoproteomic analysis of Matrigel-embedded samples. We further examined the differences between the phosphoproteome of conventional 2D-cultured samples and those of 3D-cultured samples. Finally, we applied our protocol to the phosphoproteomic analysis of patient-derived organoids from colorectal cancer.

## Results

### Removal of contaminants from Matrigel using acetone precipitation of digested peptides

**Figure 1:**
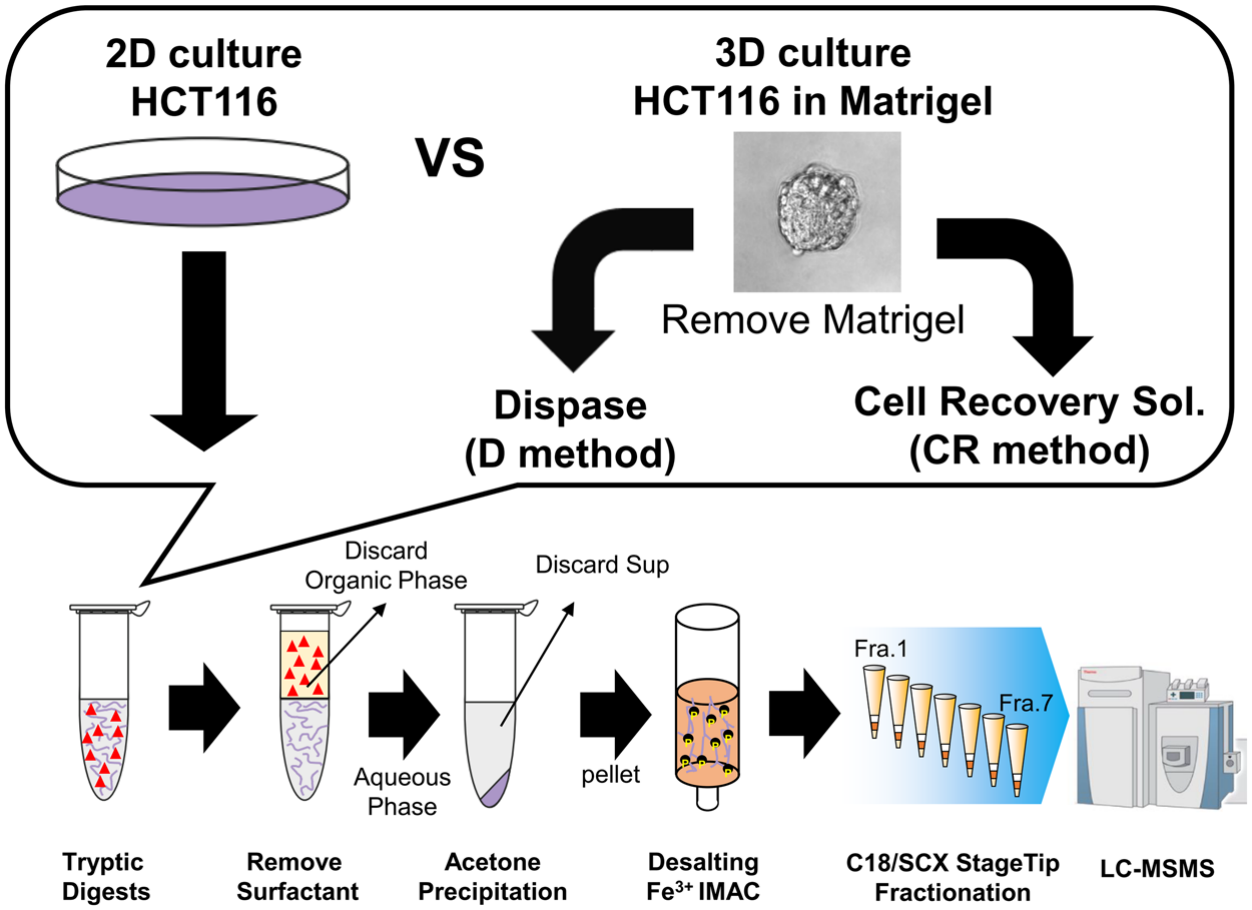
Workflow for phosphoproteomic analysis using Matrigel-embedded 3D-cultured samples purified using acetone precipitation. Red triangles: surfactants in the PTS buffer.

**Figure 2:**
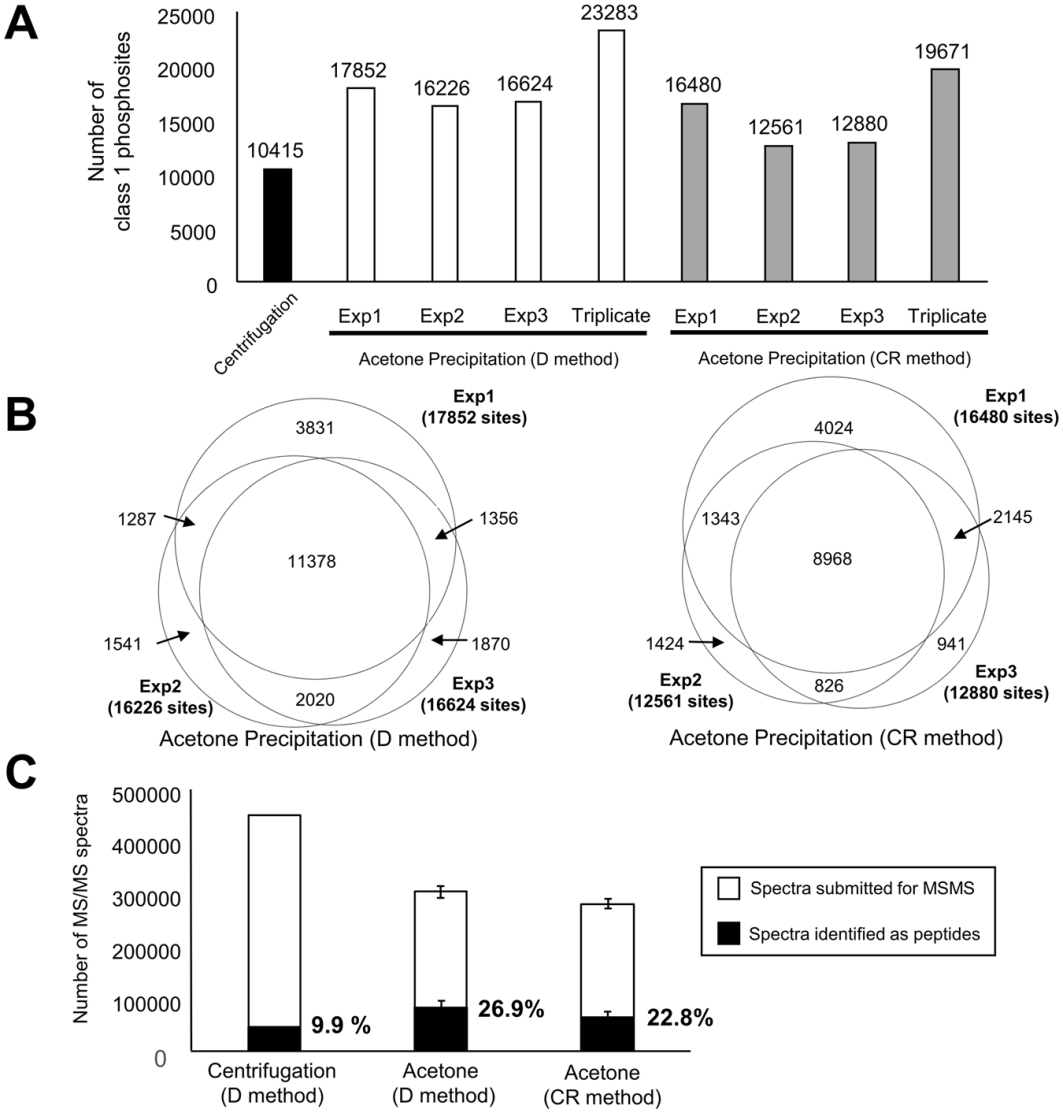
Results of phosphoproteomics from 3D HCT116 cultures embedded in Matrigel. (**A**) A bar graph of identified number of class 1 phosphosites from Matrigel-embedded HCT116 spheroid in protocol with centrifugation (a black bar) or acetone precipitation (white bars: samples collected with the D method, gray bars: samples collected with the CR method). (**B**) Venn diagrams of phosphosites identified with the D method (left panel) and the CR method (right panel). (**C**) Number of submitted MS/MS spectra (white bars) and MS/MS spectra identifying peptides (black bars). N = 3. Error bars indicate standard deviations.

In order to establish a protocol for phosphoproteomics compatible with Matrigel-embedded samples, we first sought to remove Matrigel-derived contaminants using centrifugation. As an experimental model, we used HCT116 cells cultured in Matrigel for eight days. The Matrigel-embedded HCT116 cells formed cystic spheroids (Fig. 1). We prepared phosphopeptides from 2.0 mg protein that was obtained from the HCT116 spheroids, which have been described in Fig. 1 and the Material and Methods section. Although 10,415 class 1 phosphosites are identified from the sample prepared with the centrifugation protocol (Table S1), the ion intensity and number of identified peptides per minute is significantly suppressed, as shown in the chromatographs (Fig. S1a). Thus, in order to remove contaminants such as phospholipids, we introduced acetone precipitation of tryptic peptides to the sample preparation workflow (Fig. 1). Using 2.0 mg proteins from HCT116 spheroids collected by one of two commonly used recovery methods, i.e., the Dispase method (hereafter termed D method) and Cell Recovery Solution method (hereafter termed CR method), the protocol involving acetone precipitation enabled us to identify 23,283 (D) and 19,671 (CR) class 1 phosphosites from triplicate experiments (Figs 2A,B, and Table S2). In contrast to the chromatographs of the samples prepared by the centrifugation protocol, the ion intensity in the chromatographs is significantly increased, as shown in Fig. S1b. Furthermore, we compared the number of MS/MS spectra that are identified as peptides for the centrifugation method, the D method, and the CR method. When using the centrifugation method, 9.9% of the MS/MS spectra was identified as peptides (Fig. 2C). Conversely, when using the two methods involving acetone precipitation, the average percentages are increased to 26.9% (D Method) and 22.8% (CR Method) (Fig. 2C). Together with the chromatography data, these results demonstrate that the protocol involving acetone precipitation efficiently removes contaminants that hamper ionization and the identification of phosphopeptides. Therefore, our protocol allows deeper phosphoproteomic analyses than those available through the centrifugation method.

### Experimental bias in phosphoproteomic analysis due to the procedure used for sample collection from Matrigel

Cell Recovery Solution and Dispase are commonly used for the removal of Matrigel from embedded samples. Matrigel can be depolymerized in Cell Recovery Solution at 4 °C without the use of enzymes which cause degradation to the extracellular domains of cell surfaces^13^. Alternatively, Dispase, a neutral protease from Bacillus polymyxa^14^, digests Matrigel at physiological temperature (37 °C) without any stimulation of biochemical reactions in response to low temperature. Because the cellular conditions during the removal of Matrigel are quite different between the D and the CR methods, we applied both methods to our phosphoproteomic analysis to assess whether the cellular phosphorylation status is affected by the method employed.

**Figure 3:**
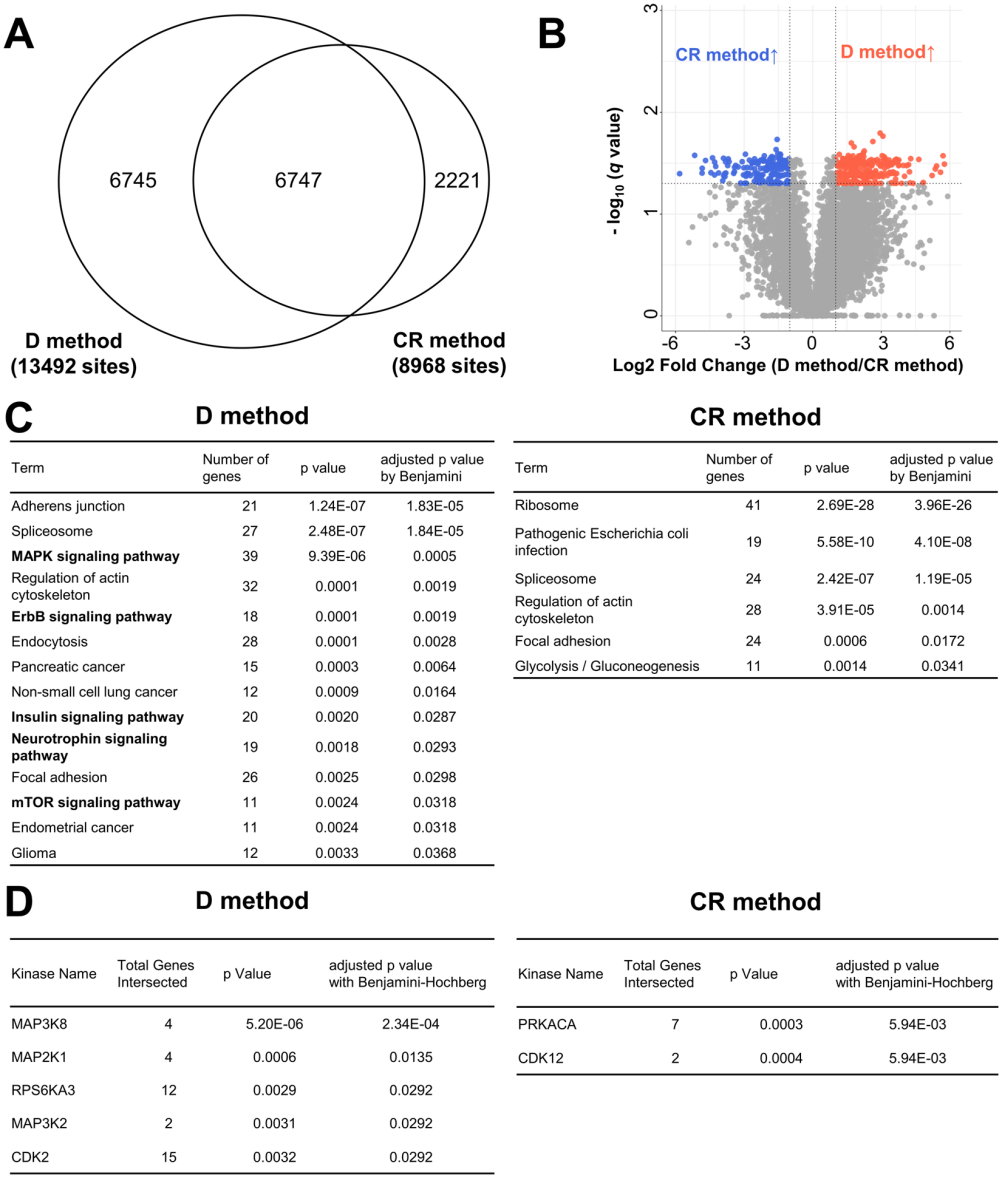
Comparison of phosphosites identified using Matrigel-embedded 3D-cultured HCT116 recovered using the D and CR methods. (**A**) Proportional Venn diagram of phosphosites identified in all triplicate experiments from Matrigel-embedded HCT116 recovered with the D and the CR method. (**B**) Volcano plot of phosphosites in comparison between samples collected with the D and CR method. (blue circles: phosphosites increased in the D method samples relative to those by the CR method; red circles: phosphosites decreased in the D method samples; gray circles: phosphosites without significant differences). (**C**) Pathway analysis results from DAVID using significantly increased or decreased phosphosites in the samples recovered with the CR method compared to those in the samples recovered with the D method (adjusted *p* value < 0.05). (**D**) List of active kinases with significant differences by KEA (adjusted *p* value < 0.05).

We compared 6,747 phosphosites identified in both experiments using Matrigel-embedded HCT116 cells recovered with the D or the CR method (Fig. 3A). Two parameters, fold change (FC) and *q* value, were used as criteria for identifying phosphorylation sites showing significant differences by D and the CR methods. Based on these criteria, we observed that 288 phosphosites in the samples using the D method are increased relative to those using the CR method, whereas 166 phosphosites were decreased in comparison to those collected using the CR method (Fig. 3B, Table S3). We further searched for the class 1 phosphosites that were identified using either one of the samples collected using the D or CR method. We observed that 1,443 and 742 phosphosites are identified only from the samples collected using the D and CR methods, respectively (Table S3).

Then, we applied these phosphosites to pathway analysis by DAVID^15^ and kinase enrichment analysis (KEA)^15,16^. In the case of the phosphosites increased or identified only from the sample in the D method, the pathway analysis revealed that several phosphosignaling pathways such as the MAPK and ErbB signaling pathways, are significantly enriched (Fig. 3C). KEA predicted significant activation of several kinases such as RPS6KA3, CDK2, and MEK1 (Fig. 3D). Activation of RPS6KA3 and CDK2 via phosphorylation itself (RPS6KA3 S78, CDK2 T160) using the D method were also confirmed by phosphoproteomic analysis (Table S3). Pathway analysis of the phosphosites enhanced by CR method did not identify activation of any phosphosignaling pathways (Fig. 3C). However, KEA analysis of the phosphosites increased or identified only from the CR method predicted activation of PRKACA and CDK12 (Fig. 3D). Coincident with the results of KEA, activation of CDK12 via phosphorylation itself (CDK12 S685) from the sample treated by the CR method was confirmed by phosphoproteomic analysis (Table S3). In summary, these data indicate that the method used for sample recovery greatly affects cellular phosphosignaling and kinome activity and can cause experimental bias in the phosphoproteomic status.

### Comparison of the phosphoproteomic statuses of differential cellular phenotypes as revealed by 2D- and 3D-cultured samples

**Figure 4:**
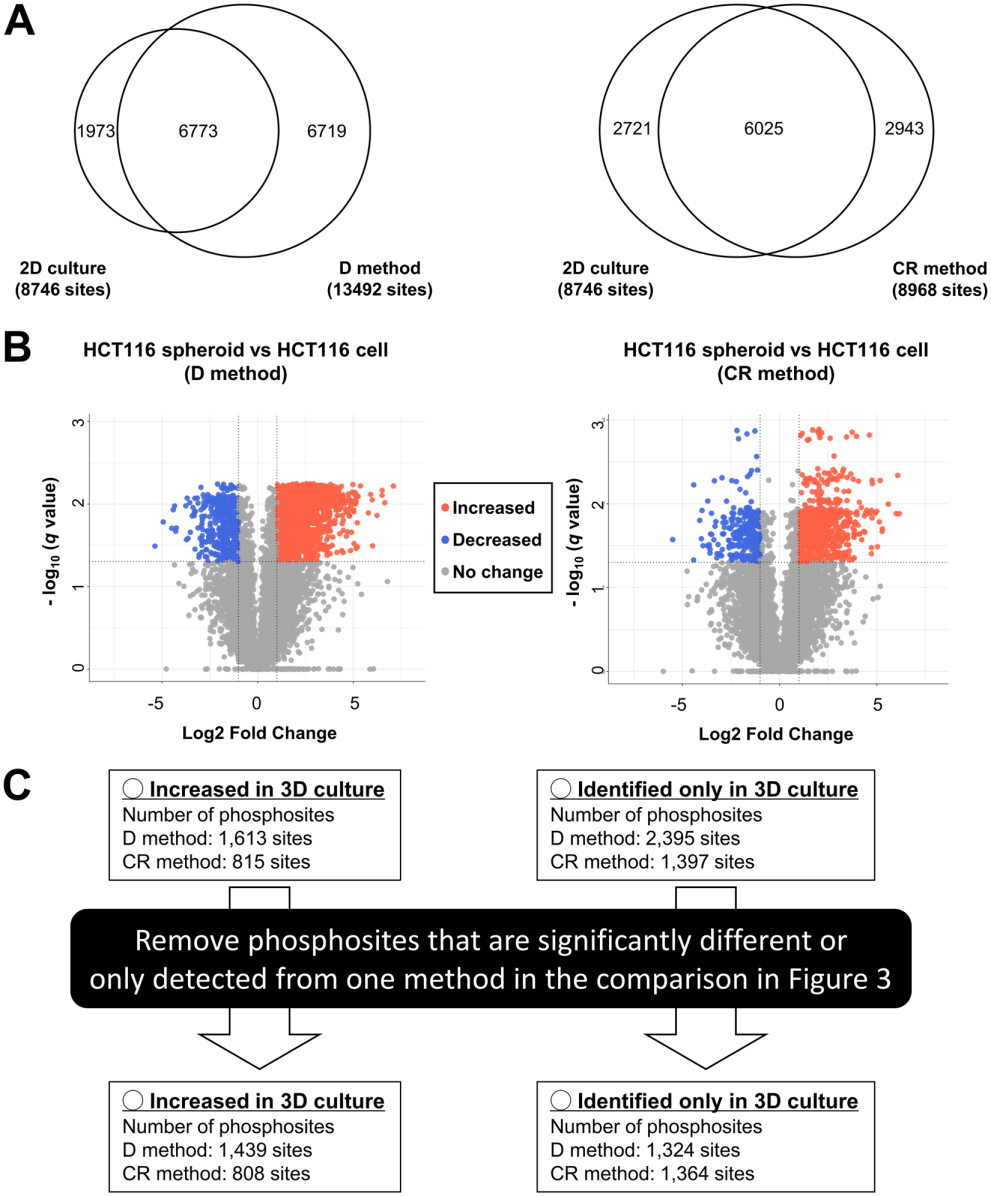
Comparison of phosphosites identified from 2D- and 3D-cultured HCT116 cells. (**A**) Proportional Venn diagram of phosphosites identified in all triplicate experiments from 2D and 3D culture of HCT116 cells. (**B**) Volcano plot of phosphosites identified in all triplicate experiments from 2D-cultured HCT116 cells and 3D cultured HCT116 spheroids. Magenta circles: increased phosphosites in the samples in the D or the CR method relative to 2D-cultured samples, Cyan circles: decreased phosphosites in the samples in the C or the CR method relative to 2D-cultured samples, Gray circles: phosphosites without significant differences. (**C**) Workflow for the selection of phosphosites that were significantly increased or identified only in the 3D-cultured samples.

3D cellular conformation is regulated through several phosphosignaling pathways^17^. Thus, we compared the phosphoproteomic statuses of 2D-cultured HCT116 cells and 3D-cultured HCT116 spheroids embedded in Matrigel to identify any modulation of phosphorylation signaling that is dependent on the 3D cellular environment. We first conducted phosphoproteomic analysis of 2D-cultured HCT116 cells with and without acetone precipitation. 15,419 class 1 phosphosites were identified from the 2D-cultured HCT116 cells with acetone precipitation (62.5% number of phosphosites being identified relative to those identified from the 2D-cultured sample without acetone precipitation) (Table S2). To investigate the difference between 2D-cultured and 3D-cultured HCT116 samples, we compared the number of phosphosites identified in all replicates using the 2D-cultured HCT116 cells with acetone precipitation to that identified using the 3D-cultured HCT116 spheroids (using either the D or CR method). As a result, of the phosphosites detected using the 2D-culture, 6,773 and 6,025 were commonly detected in all triplicates of 3D cultured samples using the D or CR methods, respectively (Fig. 4A,B). Of these 6,773 and 6,025 phosphosites, 1,613 and 815 phosphosites were significantly increased in the 3D-cultured samples recovered with the D and CR method, respectively, relative to those in the 2D-cultured samples (Fig. 4C). We also identified 2,395 and 1,397 phosphosites that were detected only in the 3D-cultured samples by the D and the CR method, respectively (Fig. 4C).

To reduce experimental bias dependent on the sample collection process as reported previously^18^, we excluded phosphosites with significant differences between the D and CR methods (listed in Table S3) from the 1,613 and the 815 phosphosites. Consequently, 1,439 and 808 phosphosites were increased in the 3D-cultured samples with the D and CR methods, respectively (Fig. 4Cand Table S4). Similarly, phosphosites only detected using either the D or the CR methods were removed from the 2,395 and 1,397 phosphosites which were identified only in the 3D-cultured samples. As a result, 1,324 and 1,364 phosphosites were identified only in the 3D-cultured samples with the D and the CR methods, respectively (Fig. 4C and Table S4).

Those phosphosites were subjected to pathway analysis and KEA to compare differences in phosphosignaling between the 3D-cultured HCT116 spheroids and 2D-cultured cells. Results from pathway analysis revealed that several phosphosignaling pathways, such as the insulin and mTOR signaling pathways, are commonly enriched in 3D-cultured cells collected by either D or CR method (Table 1). KEA predicted significant activation of MAPK9, RPS6KA3, and SGK1 under 3D conditions using both the D and the CR methods (Table 2). SGK1 is known to be associated with both the insulin and mTOR signaling pathways^19^. In addition, two phosphosites (BAG3 S289 and WNK1 S2032) assigned as mTOR substrates in PhosphositePlus^20^ were increased in both 3D samples (Table S4). Results of these phosphosites are consistent with those of the pathway analysis, further emphasizing the activation of the mTOR signaling pathway in 3D-cultured samples. Activation of RPS6KA3 via phosphorylation itself (S227) in 3D-cultured HCT116 cells was also revealed by the phosphoproteomic data from samples recovered by both methods, indicating agreement with the results of KEA analysis (Table S4). Thus, our phosphoproteomic method enables us to identify the modulation of phosphosignaling and kinome activity under 3D conditions. This information would help to rationalize the cellular conformation of 3D architectures in terms of phosphorylation-mediated regulatory mechanisms.

**Table 1:** Pathway analysis with DAVID using phosphosites that were significantly increased in the 3D culture samples compared to 2D culture samples or identified only in the 3D culture samples (adjusted *p* value < 0.05).

Term	Number of genes	*p* value	adjusted *p* value by Benjamini
**HCT116 spheroid vs HCT116 cell (D method)**			
RNA transport	48	3.38E-11	8.63E-09
Regulation of actin cytoskeleton	49	1.69E-08	2.15E-06
**Insulin signaling pathway**	36	9.21E-08	7.83E-06
**ErbB signaling pathway**	27	1.30E-07	8.30E-06
Proteoglycans in cancer	45	1.90E-07	9.71E-06
Tight junction	35	2.47E-07	1.05E-05
Endocytosis	53	2.91E-07	1.06E-05
MAPK signaling pathway	48	1.43E-05	4.55E-04
Focal adhesion	41	1.80E-05	5.09E-04
Bacterial invasion of epithelial cells	21	4.53E-05	0.0012
Pathogenic Escherichia coli infection	16	7.45E-05	0.0017
**Neurotrophin signaling pathway**	27	8.20E-05	0.0017
Insulin resistance	25	1.00E-04	0.0020
**mTOR signaling pathway**	17	1.01E-04	0.0018
Salmonella infection	21	1.16E-04	0.0020
Adherens junction	19	1.26E-04	0.0020
Fc gamma R-mediated phagocytosis	21	1.39E-04	0.0021
Thyroid hormone signaling pathway	25	2.43E-04	0.0034
**AMPK signaling pathway**	26	2.83E-04	0.0038
Epstein-Barr virus infection	35	3.91E-04	0.0050
Non-small cell lung cancer	15	8.15E-04	0.0098
**HCT116 spheroid vs HCT116 cell (CR method)**			
Ribosome	48	1.97E-18	4.78E-16
Epstein-Barr virus infection	42	7.68E-09	9.29E-07
Pathogenic Escherichia coli infection	19	5.79E-08	4.67E-06
Regulation of actin cytoskeleton	41	4.88E-07	2.95E-05
Salmonella infection	23	6.43E-07	3.11E-05
Endocytosis	46	1.10E-06	4.42E-05
**AMPK signaling pathway**	28	1.75E-06	6.06E-05
Focal adhesion	38	5.03E-06	1.52E-04
**Neurotrophin signaling pathway**	26	1.32E-05	3.54E-04
Proteoglycans in cancer	36	1.64E-05	3.96E-04
**Insulin signaling pathway**	28	2.01E-05	4.42E-04
Carbon metabolism	24	4.29E-05	8.66E-04
**mTOR signaling pathway**	16	5.39E-05	0.0010
Spliceosome	26	8.10E-05	0.0014
Protein processing in endoplasmic reticulum	30	1.25E-04	0.0020
RNA transport	30	1.71E-04	0.0026
Proteasome	13	1.73E-04	0.0025
Bacterial invasion of epithelial cells	18	1.78E-04	0.0024
**ErbB signaling pathway**	19	2.32E-04	0.0030
Endometrial cancer	14	2.44E-04	0.0029
Biosynthesis of antibiotics	34	2.97E-04	0.0034
Biosynthesis of amino acids	17	3.02E-04	0.0033
Glycolysis/Gluconeogenesis	16	3.10E-04	0.0033
Shigellosis	15	6.27E-04	0.0063
Renal cell carcinoma	15	7.40E-04	0.0071

Terms regarding phosphosignaling pathways that are commonly significant in the both 3D culture samples are indicated in bold font.

**Table 2:** Lists of active kinases predicted by KEA using phosphosites that were significantly increased in the 3D-cultured samples compared to those in the 2D-cultured samples or identified only in the 3D-cultured samples (adjusted *p* value < 0.05).

HCT116 spheroid vs HCT116 cell (D method)				HCT116 spheroid vs HCT116 cell (CR method)			
Kinase Name	Total Genes Intersected	*p* Value	adjusted *p* value with Benjamini-Hochberg	Kinase Name	Total Genes Intersected	p Value	adjusted p value with Benjamini-Hochberg
CDK1	47	3.73E-07	3.06E-05	**RPS6KA3**	35	5.78E-13	4.57E-11
CDK2	45	8.55E-07	3.06E-05	PRKACA	21	8.3E-06	3.26E-04
**MAPK9**	19	9.99E-07	3.06E-05	AKT1	14	3.5E-05	9.24E-04
GSK3B	55	2.24E-06	5.15E-05	PRKCD	9	0.00015	3.04E-03
MAPK14	39	8.70E-06	1.60E-04	**SGK1**	7	0.00054	8.59E-03
**RPS6KA3**	32	2.60E-05	3.99E-04	SGK3	4	0.00126	0.0166
MAPK10	13	0.0008	0.0105	**MAPK9**	9	0.0025	0.0283
PAK4	4	0.0021	0.0229	CDK17	2	0.00395	0.039
PDK1	7	0.0022	0.0229				
SGK1	8	0.0030	0.028				

Kinase names that are commonly significant in both the 3D-cultured samples are indicated in bold font.

### Global phosphoproteomic analysis using patient-derived organoids by fractionated or one-shot proteomic methods

The application of organoid systems has been rapidly extending to various types of cancers, including prostate cancer, pancreatic cancer, and hepatocellular carcinoma^6–8,21^. Although personal omics profiling with patient-derived organoids has steadily progressed^7^, research into phosphoproteomic profiling from patient-derived organoids is still lacking owing to the technical challenges associated with sample preparation.

**Figure 5:**
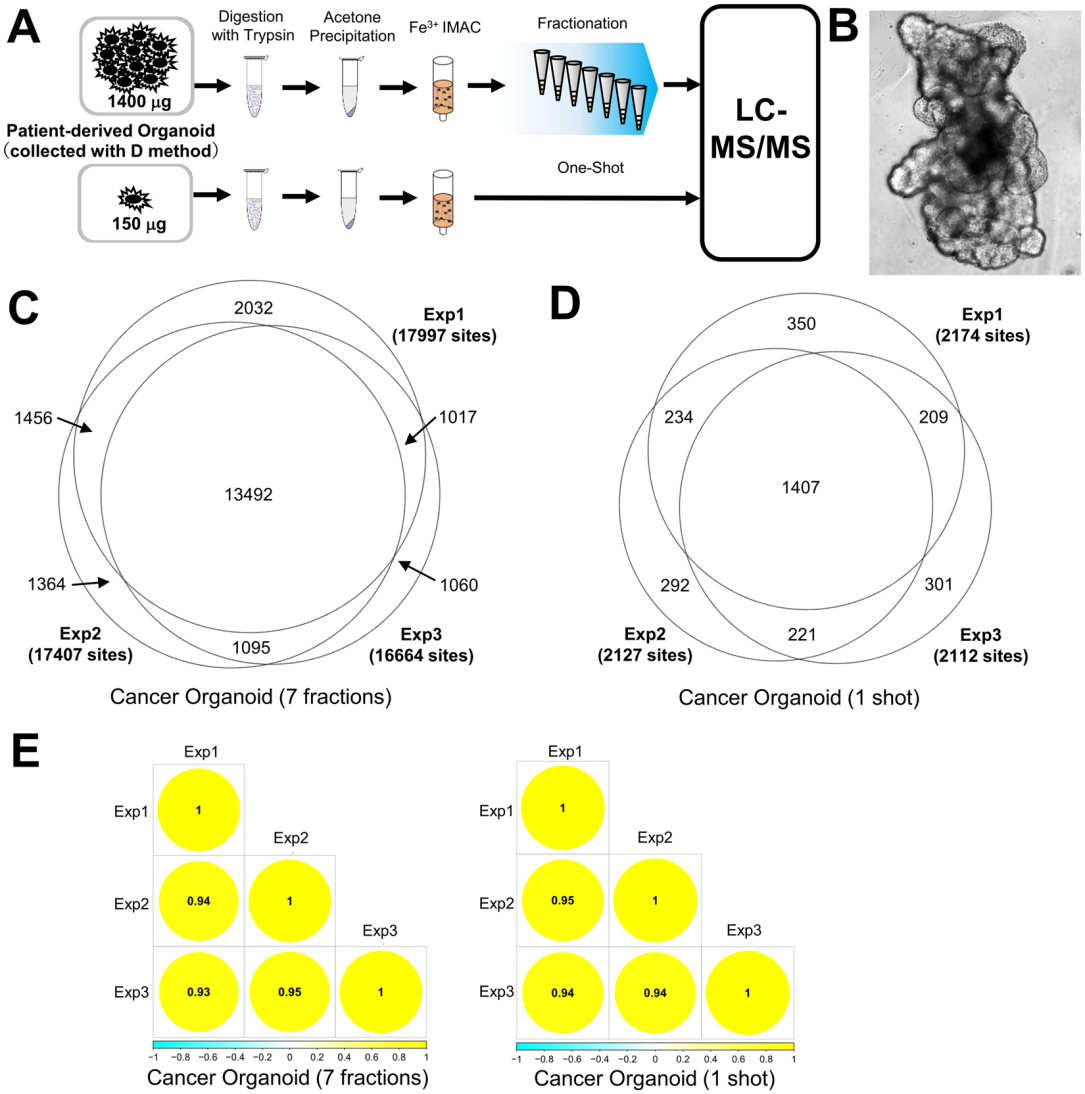
Large-scale and small-scale phosphoproteomics using patient-derived organoids. (**A**) Workflow of large-scale and small-scale phosphoproteomics. (**B**) Picture of a patient-derived organoid. (**C**) Proportional Venn diagram of class 1 phosphosites identified from patient-derived organoids with fractionation. (**D**) Proportional Venn diagram of class 1 phosphosites identified from patient-derived organoids in a one-shot analysis. (**E**) Correlation matrix of phosphosites quantified in all triplicate fractionated and one-shot phosphoproteomics experiments.

Thus, we sought to determine whether our protocol is applicable to phosphoproteomic analysis using patient-derived organoids at the large scale (1.4 mg) and small scale (150 μg). Patient-derived organoids were collected with the D method because a higher number of phosphosites were identified in HCT116 spheroids by the D method relative to that by the CR method (Fig. 5A). Patient-derived organoids show crypt-like structures that resemble the 3D architecture of colorectal cancer tissue (Fig. 5B)^7,22^. First, we collected 1.4 mg (samples from 32 wells in 48 well-plate) of protein lysate from patient-derived organoids. In total, 21,516 phosphopeptides were identified from triplicate experiments (Fig. 5C and Table S5), indicating that our protocol enables phosphoproteomic analysis of patient-derived organoids at equal sensitivity to that achieved for cultured cell lines.

To apply proteomics to precision medicine, it is essential to acquire comprehensive data from a small amount of patient-derived sample in an expeditious manner. Therefore, for the purpose of high-throughput phosphoproteomics from patient-derived organoids, we attempted a small-scale phosphoproteomic analysis without peptide fractionation before LC-MS/MS. We collected 150 μg of protein lysate from patient-derived organoids (4 wells in a 48 well-plate). This one-shot phosphoproteomic analysis resulted in identification of 2,979 phosphopeptides from triplicate experiments (Fig. 5D and Table S5). Subsequently, we evaluated the quantitative reproducibility of the phosphosites data from the large-scale (fractionated) and small-scale (one-shot) analyses. Pearson’s correlation coefficients for the one-shot phosphoproteomic analysis (0.93 to 0.95) were virtually equivalent to those of the fractionated phosphoproteomic analysis (0.94 to 0.95) (Fig. 5E). This result demonstrates the excellent reproducibility available with our phosphoproteomic protocol, even for small-scale samples.

Thus, these data indicate that our protocol could be applied for obtaining global phosphoproteomics data from patients-derived organoids.

## Discussion

In this study, we examined acetone precipitation for phosphoproteomic analysis of Matrigel-embedded samples in order to reduce contaminants from Matrigel. We obtained global phosphoproteomic data from Matrigel-embedded spheroids and patient-derived organoids.

In previous studies, precipitation of proteins with acetone, acetone/trichloroacetic acid, and methanol/chloroform has been reported to be utilized for the elimination of contaminants^12^. However, in this study, we have applied the precipitation of digested peptides, and not proteins, by adding acetone and have achieved a significant decrease in contaminants and identification of global phosphoproteomics (Fig. 2). These results indicate that our protocol may be potentially applied in case of peptidome using samples that contain large amount of contaminants such as plasma^23^ and neoantigen in the cancer tissue^24^. Additionally, a previous report demonstrated the selective precipitation of phosphopeptides using Ba^2+^/acetone from whole digested peptides^25^. Therefore, our protocol may be further applied to not only eliminate contaminants but also to selectively enrich the digested peptides without the usage of immunoprecipitation.

It has been reported that alteration of phosphoproteomic data can be caused by processes during sample collection, such as ischemia in surgery and washing before lysis^18,26^. Similarly, in this study, we have observed that phosphoproteomic data is biased dependent on the method used for the removal of the Matrigel. Pathway analysis indicted that several phosphosignals are activated in the samples isolated by the D method compared to those by the CR method. Although the number of phosphosites identified is higher using the D method than that using the CR method, the CR method is a better way to harvest Matrigel-embedded samples in terms of minimizing the fluctuation of phosphosignaling during sample collection. Recently, the stiffness of the scaffold has been reported to be important for efficient expansion of organoids^27^. Accordingly, the results of this study indicate that our protocol allows phosphoproteomic analysis of samples embedded in such hard conditions. Further improvements of the current protocol are expected to minimize experimental bias during sample collection, and to enable phosphoproteomic analysis of organoids cultured in much stiffer scaffolds.

3D cancer models have been utilized to investigate the mechanisms of cancer growth and invasion in conditions closer to *in vivo* environments. For example, previous studies with 3D cancer spheroids have demonstrated the differential relationship between extracellular conditions and intracellular signaling pathways in malignant cancer and normal tissues^17^. In the current study, phosphoproteomic analysis of 3D-cultured HCT116 spheroids revealed the activation of several phosphosignaling pathways, such as the AMPK, mTOR, and ErbB pathways (Table 1). Because the different results obtained from the 2D and 3D culture condition were not caused by methodological bias (Fig. 4), we concluded that the signaling pathways were activated because of the difference between the culture conditions that were observed in the 2D and 3D circumstances. Collectively, these results support the conclusions of previous studies, i.e., that those pathways are associated with the 3D growth and metastasis of malignant cancer^28–30^. This fact indicates that our approach has the potential to reveal the precise modulation of phosphosignaling in cancers under 3D conditions. Therefore, our protocol for Matrigel-embedded samples would contribute comprehensive understanding of phosphorylation-mediated regulation in 3D behavior of cancer.

In recent years, methods for high-throughput phosphoproteomics using relatively small amounts (<1 mg) of protein lysate have been developed, such as the EasyPhos technique, which can identify more than 10,000 phosphosites from 1 mg of protein lysate^31^. In combination with pharmacological response data, quick and sensitive phosphoproteomic analysis could facilitate the prediction of drug sensitivity and provide important details of phosphosignaling under drug treatment. In the current study, our method enabled us to identify almost 3,000 phosphosites from 150 μg lysate of patient-derived organoids (Fig. 5C). The combination of our protocol with multiplex labeling techniques may provide further improvement to deep and high-throughput phosphoproteomics.

In summary, we have achieved the accurate identification of cellular phosphorylation characteristics not only from large-scale samples, but also from small scale samples. Therefore, the phosphoproteomic method developed in this study may contribute significantly to the profiling of individual phosphorylation networks in cancer patients from limited amount of patient derived samples such as organoids. Furthermore, our results indicate that our phosphoproteomic procedure represents a significant breakthrough for providing novel insights into the 3D architectures of cancers, thus facilitating the identification of novel druggable targets.

## Material and Methods

### Reagents

Dispase, Cell Recovery Solution, Penicillin-streptomycin, and fetal bovine serum (FBS) were obtained from Thermo Fisher Scientific (Waltham, MA, USA). Oasis HLB cartridges were purchased from Waters (Milford, MA, USA). A detergent-compatible (DC) protein assay kit was obtained from Bio-Rad (Hercules, CA, USA). Chemi-Lumi Super and Dulbecco’s modified Eagle’s medium (DMEM) were purchased from Nacalai Tesque (Kyoto, Japan). Phosphate-buffered saline (PBS) tablets were obtained from Takara (Shiga, Japan). cOmplete protease inhibitor cocktail, PhosSTOP phosphatase inhibitor cocktail, and trypsin were obtained from Roche (Basel, Switzerland).

### Cell cultures and sample collection

HCT116 cells were maintained in DMEM supplemented with 10% FBS and 1% Penicillin-streptomycin at 37 °C under 5% CO_2_. The Matrigel-embedded cultures used in the current study were prepared by mixing 4,000 HCT116 cells with 25 μL of ice-cold Matrigel followed by mounting in a 48-well plate. After incubation for 10 min at 37 °C, DMEM supplemented with FBS and penicillin-streptomycin was overlaid. Patient-derived organoids were established from surgically resected tumor samples at the JFCR Cancer Institute (Tokyo, Japan). Informed consent was obtained from all donors. The experimental protocol was approved by the ethics committees of the National Institute of Biomedical Innovation Health and Nutrition, and the JFCR Cancer Institute. All methods were carried out according to relevant guidelines and regulations. Organoids were cultured as previously described^21^. Based on the previous study, organoids were derived only from the malignant part of the surgical cancer tissue. The culture medium was exchanged with fresh medium every two days. HCT116 cells and patient-derived organoids were collected after washing with ice-cold PBS buffer containing PhosSTOP and cOmplete and lysed in phase transfer surfactant (PTS) buffer (12 mM sodium deoxycholate, 12 mM sodium lauroyl sarcosinate, 50 mM ammonium bicarbonate)^32^. Lysates were boiled at 95 °C for 5 min followed by ultrasonication three times by using a Bioruptor sonicator (Cosmo Bio, Tokyo, Japan) for 15 min. The matrigel-embedded HCT116 and patient-derived organoids were completely solubilized under the condition. The Matrigel-embedded HCT116 spheroids and patient-derived organoids were harvested at eight days post-passage. When collected with Dispase, Matrigel-embedded samples were washed once with 1x PBS and mixed with 200 μL of 50 unit/ml Dispase solution. After incubation at 37 °C for 2 h, digestion with Dispase was quenched with 800 μL of 10 mM EDTA. The samples were then washed twice with 1x PBS, and dissolved in PTS buffer. For sample collection with Cell Recovery Solution (the composition of the solution has not been disclosed by the vendor), the samples were washed three times with 1x PBS, and mixed with 1 mL of Cell Recovery Solution. The mixture was incubated on ice for 1 h then washed twice with 1x PBS and dissolved in PTS buffer. Cell lysates were flash-frozen with liquid nitrogen and stored at −80 °C.

### Digestion with trypsin, removal of surfactant, and acetone precipitation of tryptic peptides

After measuring the protein concentration of the cell lysate with a DC protein assay, the lysate samples were reduced, alkylated, and subsequently trypsinized as described previously^33^. The surfactant was then removed as also described previously^32^. Briefly, the proteins that were dissolved in the PTS buffer were digested with trypsin that was followed by vigorous mixing with an equal volume of ethyl acetate containing 1% trifluoroacetic acid. Further, centrifugation at 14,000 g was conducted for 3 min at 4 °C. The surfactant in the PTS buffer was transferred to an organic phase, and the aqueous phase of the mixture was subjected to the following centrifugation or acetone precipitation step. For the centrifugation protocol, the aqueous phase containing tryptic peptides was centrifuged at 12,000 g for 5 min. For the acetone precipitation protocol, the pelleted peptides were mixed with 1 mL of ice-cold acetone, sonicated for 10 min, and stored at −20 °C for 2 h. Then, the tryptic peptides were centrifuged at 12,000 g for 5 min. After discarding the supernatant, the samples were lyophilized for 10 min. The pellets were resuspended in 2 M urea and 1% TFA solution and subjected to desalination using an OASIS HLB column.

### Enrichment of phosphopeptides, TMT labeling, and fractionation with C18/SCX stage-tips

Enrichment of phosphopeptides with Fe^3+^ IMAC resin was performed as described previously^34^. Phosphopeptides were divided into seven fractions with C18-SCX stage-tips as described previously^35^. For the one-shot phosphoproteomics, desalting with C18 stage-tips was conducted after phosphopeptide enrichment.

### Mass spectrometry analysis

Equipment for LC-MS/MS analysis was corresponding to a previous study^36^. The nano-LC gradient was performed at 280 nL/min and consisted of a linear gradient of buffer B from 5 to 30% B over 135 min. Parameters in Q Exactive instrument was coincident with the condition in the previous study^36^.

### Data processing for identification and quantification of peptides

Phosphopeptide identification was conducted with MaxQuant 1.5.1.2 supported by the Andromeda search engine^37^. The UniProt human database (release 2011_11) combined with 262 common contaminants was used for the analysis of MS/MS spectra. Enzyme specificity was set to a C-terminal of Arg or Lys with the allowed cleavage at the proline bond. Up to two miss-cleavages were tolerated. Fixed modification was performed by carbamidomethylation of cysteine residues. Variable modifications were performed by methionine oxidation and/or serine, threonine, and tyrosine phosphorylation. Protein group, peptide, and PTM site levels with <0.01 FDR were accepted. Peptides annotated as “Reverse” or “Potential Contaminant” were omitted. The cut-off criteria for phosphopeptides were those used in a previous study^38^. Details for the identification of phosphopeptides are described below. Phosphorylated and nonphosphorylated peptides are distinguished by the mass shift corresponding to the modification of phosphorylation (molecular weight = 79.966) at the MS1 level. Additionally, the MS2 scans (fragmented ionic products of phosphopeptides) are utilized to determine the localization of phosphorylation in the sequence of phosphopeptides^39^. Further, phosphopeptides with highly reliable identification were extracted and subjected to further analysis. The criteria of phosphopeptides included an Andromeda delta score >8 and class 1 phosphosites (localization probability >0.75). “Class 1 phosphosite” was a qualitative standard for phosphoproteomics that have been defined in a previous study^40^.

### Statistical analysis of identified class 1 phosphosites

Statistical analysis was carried out with Perseus 1.5.5.3 (www.perseus-framework.org)^41^. The data in label-free quantification were log_2_ transformed and normalized using the sum of all intensities and median centering of the values in each sample. *p* values were calculated with the two-tailed welch *t* test, then adjusted to a *q* value with the permutation test. Fold change and *q* value were used for eliminating phosphosites that showed significant differences. The cutoff criteria for fold change were more than twice or less than half that of a control. Additionally, the phosphosites with a *q* value lower than 0.05 were used for subsequent analysis. Proportional Venn diagrams were prepared with eulerAPE^42^. The correlation matrix was constructed with the use of the R package “Correlation Matrix.” Pathway analysis of the extracted phosphosites was performed with KEGG in DAVID^15^. KEA ver. 2 was employed according to the reported protocol^16^.

### Data availability

Accession code of MS data in this study is PXD009032 in jPOST (http://jpostdb.org/)^43^.

## Electronic supplementary material

Supplementary information

Supplementary dataset1

Supplementary dataset2

Supplementary dataset3

Supplementary dataset4

Supplementary dataset5

## References

[1] Thoma CR, Zimmermann M, Agarkova I, Kelm JM, Krek W (2014). 3D cell culture systems modeling tumor growth determinants in cancer target discovery. Advanced drug delivery reviews.

[2] Nath S, Devi GR (2016). Three-dimensional culture systems in cancer research: Focus on tumor spheroid model. Pharmacology & therapeutics.

[3] Kretzschmar K, Clevers H (2016). Organoids: Modeling Development and the Stem Cell Niche in a Dish. Developmental cell.

[4] Lancaster MA, Knoblich JA (2014). Organogenesis in a dish: modeling development and disease using organoid technologies. Science.

[5] Sachs N, Clevers H (2014). Organoid cultures for the analysis of cancer phenotypes. Current opinion in genetics & development.

[6] Gao D (2014). Organoid cultures derived from patients with advanced prostate cancer. Cell.

[7] van de Wetering M (2015). Prospective derivation of a living organoid biobank of colorectal cancer patients. Cell.

[8] Boj SF (2015). Organoid models of human and mouse ductal pancreatic cancer. Cell.

[9] Fleuren ED, Zhang L, Wu J, Daly RJ (2016). The kinome ‘at large’ in cancer. Nature reviews. Cancer.

[10] Abe Y (2017). Deep Phospho- and Phosphotyrosine Proteomics Identified Active Kinases and Phosphorylation Networks in Colorectal Cancer Cell Lines Resistant to Cetuximab. Scientific reports.

[11] Zanivan S (2013). SILAC-based proteomics of human primary endothelial cell morphogenesis unveils tumor angiogenic markers. Molecular & cellular proteomics: MCP.

[12] Feist P, Hummon AB (2015). Proteomic challenges: sample preparation techniques for microgram-quantity protein analysis from biological samples. International journal of molecular sciences.

[13] Rana B, Mischoulon D, Xie Y, Bucher NL, Farmer SR (1994). Cell-extracellular matrix interactions can regulate the switch between growth and differentiation in rat hepatocytes: reciprocal expression of C/EBP alpha and immediate-early growth response transcription factors. Molecular and cellular biology.

[14] Stenn KS, Link R, Moellmann G, Madri J, Kuklinska E (1989). Dispase, a neutral protease from Bacillus polymyxa, is a powerful fibronectinase and type IV collagenase. The Journal of investigative dermatology.

[15] Huang da W, Sherman BT, Lempicki RA (2009). Systematic and integrative analysis of large gene lists using DAVID bioinformatics resources. Nature protocols.

[16] Lachmann A, Ma’ayan A (2009). KEA: kinase enrichment analysis. Bioinformatics.

[17] Yamada KM, Cukierman E (2007). Modeling tissue morphogenesis and cancer in 3D. Cell.

[18] Mertins P (2014). Ischemia in tumors induces early and sustained phosphorylation changes in stress kinase pathways but does not affect global protein levels. Molecular & cellular proteomics: MCP.

[19] Laplante M, Sabatini DM (2012). mTOR signaling in growth control and disease. Cell.

[20] Hornbeck PV (2015). PhosphoSitePlus, 2014: mutations, PTMs and recalibrations. Nucleic acids research.

[21] Sato T (2011). Long-term expansion of epithelial organoids from human colon, adenoma, adenocarcinoma, and Barrett’s epithelium. Gastroenterology.

[22] Fujii M (2016). A Colorectal Tumor Organoid Library Demonstrates Progressive Loss of Niche Factor Requirements during Tumorigenesis. Cell stem cell.

[23] Liotta LA, Petricoin EF (2006). Serum peptidome for cancer detection: spinning biologic trash into diagnostic gold. The Journal of clinical investigation.

[24] Polyakova A, Kuznetsova K, Moshkovskii S (2015). Proteogenomics meets cancer immunology: mass spectrometric discovery and analysis of neoantigens. Expert review of proteomics.

[25] Ruse CI (2008). Motif-specific sampling of phosphoproteomes. Journal of proteome research.

[26] Kanshin E, Tyers M, Thibault P (2015). Sample Collection Method Bias Effects in Quantitative Phosphoproteomics. Journal of proteome research.

[27] Gjorevski N (2016). Designer matrices for intestinal stem cell and organoid culture. Nature.

[28] Wilson SM (2008). mTOR mediates survival signals in malignant mesothelioma grown as tumor fragment spheroids. American journal of respiratory cell and molecular biology.

[29] Yu M (2012). Expression profiling during mammary epithelial cell three-dimensional morphogenesis identifies PTPRO as a novel regulator of morphogenesis and ErbB2-mediated transformation. Molecular and cellular biology.

[30] Peart T (2015). Intact LKB1 activity is required for survival of dormant ovarian cancer spheroids. Oncotarget.

[31] Humphrey SJ, Azimifar SB, Mann M (2015). High-throughput phosphoproteomics reveals *in vivo* insulin signaling dynamics. Nature biotechnology.

[32] Masuda T, Tomita M, Ishihama Y (2008). Phase transfer surfactant-aided trypsin digestion for membrane proteome analysis. Journal of proteome research.

[33] Adachi J (2014). Proteome-wide discovery of unknown ATP-binding proteins and kinase inhibitor target proteins using an ATP probe. Journal of proteome research.

[34] Matsumoto M (2009). Large-scale proteomic analysis of tyrosine-phosphorylation induced by T-cell receptor or B-cell receptor activation reveals new signaling pathways. Proteomics.

[35] Adachi J (2016). Improved Proteome and Phosphoproteome Analysis on a Cation Exchanger by a Combined Acid and Salt Gradient. Analytical chemistry.

[36] Abe Y, Nagano M, Tada A, Adachi J, Tomonaga T (2017). Deep Phosphotyrosine Proteomics by Optimization of Phosphotyrosine Enrichment and MS/MS Parameters. Journal of proteome research.

[37] Cox J, Mann M (2008). MaxQuant enables high peptide identification rates, individualized p.p.b.-range mass accuracies and proteome-wide protein quantification. Nature biotechnology.

[38] Sharma K (2014). Ultradeep human phosphoproteome reveals a distinct regulatory nature of Tyr and Ser/Thr-based signaling. Cell reports.

[39] Chalkley RJ, Clauser KR (2012). Modification site localization scoring: strategies and performance. Molecular & cellular proteomics: MCP.

[40] Olsen JV (2006). Global, *in vivo*, and site-specific phosphorylation dynamics in signaling networks. Cell.

[41] Tyanova S (2016). The Perseus computational platform for comprehensive analysis of (prote)omics data. Nature methods.

[42] Micallef L, Rodgers P (2014). eulerAPE: drawing area-proportional 3-Venn diagrams using ellipses. Plos one.

[43] Okuda S (2017). jPOSTrepo: an international standard data repository for proteomes. Nucleic acids research.

