# Supplementary material for: Improved phosphoproteomic analysis for phosphosignaling and active-kinome profiling in Matrigel-embedded spheroids and patient-derived organoids
Source: Sci Rep. 2018 Jul 30;8:11401. doi: 10.1038/s41598-018-29837-1 (PMC6065387; doi:10.1038/s41598-018-29837-1)

# "Improved phosphoproteomic analysis for phosphosignaling and active-kinome profiling in Matrigel-embedded spheroids and patient-derived organoids."

Yuichi Abe[1,2], Asa Tada[1,2], Junko Isoyama[1,2], Satoshi Nagayama[3], Ryoji Yao[4], Jun Adachi[1,2], Takeshi Tomonaga*[1,2]

[1] Laboratory of Proteome Research, National Institute of Biomedical Innovation, Health and Nutrition, Ibaraki, Osaka 567-0085, Japan,[2] Laboratory of Proteomics for Drug Discovery, Center for Drug Design Research, National Institute of Biomedical Innovation, Health and Nutrition, Ibaraki, Osaka 567-0085, Japan,[3] Department of Gastroenterological Surgery, Cancer Institute Hospital, Japanese Foundation for Cancer Research, 135-8550, Tokyo, Japan,[4] Division of Cell Biology, Cancer Institute, Japanese Foundation for Cancer Research, 135-8550, Tokyo, Japan

**Table of Contents**

**Figure S1** Total ion chromatogram of phosphoproteomic analysis.

**Table S1** Summary of the class 1 phosphorylation sites detected in an analysis of HCT16 samples with the "Centrifugation" protocol.

**Table S2** Summary of the class 1 phosphorylation sites detected at least one time of the triplicate experiments in 2D-cultured HCT116 cells samples (with or without acetone precipitation) or Matrigel-embedded HCT116 spheroids collected with Dispase or Recovery solution.

**Table S3** Summary of phosphorylation sites showing significant differences between HCT116 spheroids collected with Dispase and Recovery solution.

**Table S4** Summary of phosphorylation sites showing significant differences between 2D-cultured HCT116 cells and Matrigel-embedded HCT116 spheroids.

**Table S5** Summary of the class 1 phosphorylation sites detected at least one time of the triplicate experiments in one-shot or fractionate phosphoproteomics of patient-derived organoids.

**Figure S1** Total ion chromatogram of phosphoproteomic analysis. Chromatograms of samples prepared with centrifugation protocol (Figure S1A) and acetone precipitation protocol (Figure S1B) were shown. Red dotted lines indicate number of identified phosphopeptides per minute.

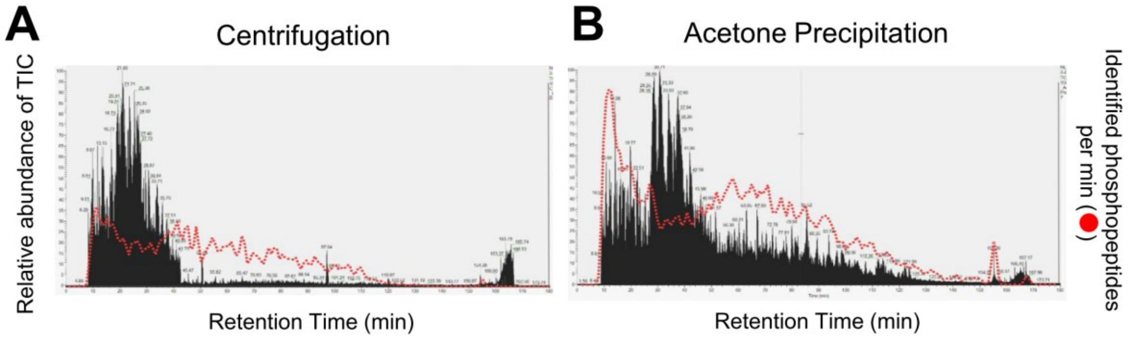

Supplement: Supplementary file 1 — Supplementary information [file 41598_2018_29837_MOESM1_ESM.pdf]
